# Toothbrushing Systematics Index (TSI) – A new tool for quantifying systematics in toothbrushing behaviour

**DOI:** 10.1371/journal.pone.0196497

**Published:** 2018-04-30

**Authors:** Nadine Schlueter, Katrin Winterfeld, Vicenç Quera, Tobias Winterfeld, Carolina Ganss

**Affiliations:** 1 Division for Cariology, Department of Operative Dentistry and Periodontology, Medical Center—University of Freiburg, Faculty of Medicine, University of Freiburg, Freiburg, Germany; 2 Department of Conservative and Preventive Dentistry, Dental Clinic of the Justus-Liebig-University Giessen, Giessen, Germany; 3 Institute of Neurosciences, Quantitative Psychology Unit, Faculty of Psychology, University of Barcelona, Barcelona, Spain; Menzies Health Institute Queensland, Griffith University, AUSTRALIA

## Abstract

Systematics is considered important for effective toothbrushing. A theoretical concept of systematics in toothbrushing and a validated index to quantify it using observational data is suggested. The index consists of three components: completeness (all areas of the dentition reached), isochronicity (all areas brushed equally long) and consistency (avoiding frequent alternations between areas). Toothbrushing should take a sufficient length of time; therefore, this parameter is part of the index value calculation. Quantitative data from video observations were used including the number of changes between areas, number of areas reached, absolute brushing time and brushing time per area. These data were fed into two algorithms that converted the behaviour into two index values (each with values between 0 and 1) and were summed as the Toothbrushing Systematics Index (TSI) value; 0 indicates completely unsystematic and 2 indicates perfectly systematic brushing. The index was developed using theoretical data. The data matrices revealed the highest values when all areas are reached and brushed equally long. Few changes occurred between the areas when the brushing duration was ≥90 s; the lowest values occurred under opposite conditions. Clinical applicability was tested with data from re-analysed videos from an earlier intervention study aiming to establish a pre-defined toothbrushing sequence. Subjects who fully adopted this sequence had a baseline TSI of 1.30±0.26, which increased to 1.74±0.09 after the intervention (p≤0.001). When the participants who only partially adopted the sequence were included, the respective values were 1.25±0.27 and 1.69±0.14 (p≤0.001). The suggested new TS-index can cover a variety of clinically meaningful variations of systematic brushing, validly quantifies the changes in toothbrushing systematics and has discriminative power.

## Introduction

**Fig 1:**
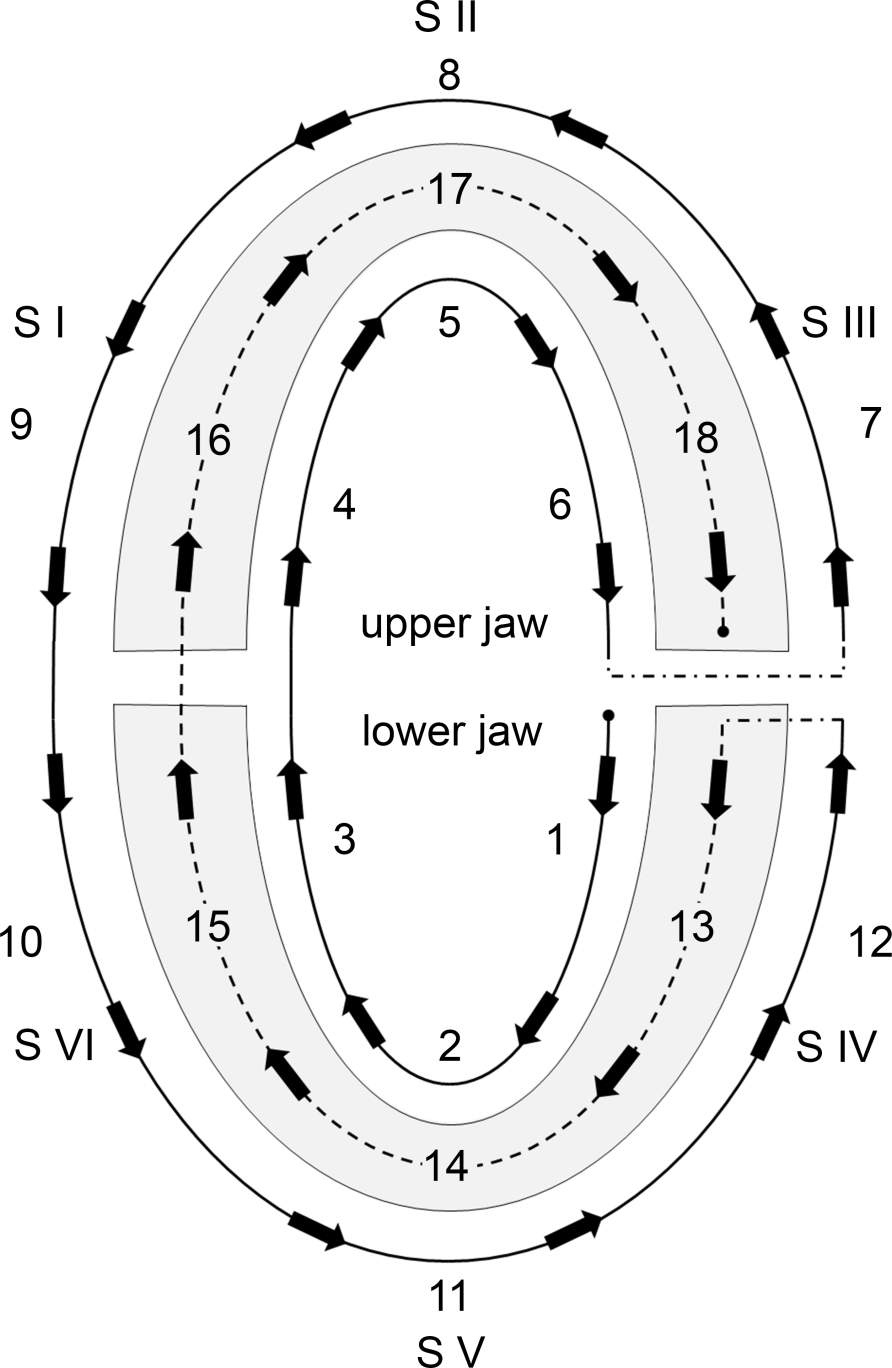
Brushing systematics according to Rateitschak [3]. The sextants (S I—S VI) and the surfaces (oral vestibular, occlusal) should be reached in a defined order, as indicated by the numbers and arrows. The starting point in this systematics is the oral surface in S IV and it ends on the occlusal surface in S III.

Toothbrushes are the most established tools for plaque control [1], and the best practices of how to use them have been widely investigated. Amongst others factors, brushing duration, technique and frequency have been evaluated and there is a great diversity in the recommendations related to these factors [2]. However, only rarely addressed is a much more basic need for proper toothbrushing; all regions of the dentition should be sufficiently cleaned. To achieve this need, brushing should be performed in a systematic and structured manner. One suggestion for such brushing systematics has been published by Rateitschak (Fig 1 [3]), who suggested that brushing should start on posterior oral surfaces of the lower jaw, on the right side for right-handers (vice versa for left-handers), continue to the anterior and the contra lateral posterior region, then to the oral surfaces of the upper jaw moving from posterior to anterior and to the contra lateral posterior sites. Then, the vestibular sites should be brushed in a similar order, until the occlusal aspects are finally addressed.

Taking the Rateitschak systematic as an example, three components essential for systematic brushing can be deduced: (a) completeness (all areas of the dentition should be reached) (b) isochronicity (equal distribution of brushing duration between all areas of the dentition), and (c) consistency (avoiding frequent alternations between areas). Furthermore, toothbrushing should take enough time to be effective.

Thus, however, the role of systematic brushing and how and whether it is achieved e.g. through oral hygiene instructions, has been rarely addressed in the literature, even though it seems plausible that it is an effective predictor of efficient plaque removal.

Video observation studies have consistently shown that subjects rarely demonstrate systematic brushing; instead, most brush predominantly on vestibular surfaces and with frequent changes between the anterior and the lateral aspects of the dentition, while neglecting the oral surfaces [4–6]. These findings emphasise the need for more research on oral hygiene behaviour and instruction particular in terms of systematics.

Off the few studies that have observed habitual brushing behaviour [7–12], two scored systematic brushing when a participant brushed either from one side to the other in each jaw or on the buccal, lingual, and occlusal surfaces from one side to the other, and classified the subjects as brushing systematically simply in crude yes/no categories [7,8]. Two other detailed studies investigated whether a precisely pre-defined course of the toothbrush was adopted after instruction [11,12]. However, these evaluation criteria are very strict and do not address clinically meaningful deviations from a given brushing order. Therefore, to date, there is no meaningful methodological solution for measuring or quantifying a whole brushing systematics or at least the components of a brushing systematics.

Therefore, the aim of the present study was to develop an index system that allows for the differentiated assessment of brushing behaviour by considering all components of brushing systematics (completeness, isochronicity and consistency) independent from predefined movement patterns. To achieve this goal, a theoretical concept for a toothbrushing systematics index (TSI) was developed, and the concept was tested using theoretical data in a first step. In a second step, re-analysed video recordings from a previous study [11] were used to validate the new index, with clinical data.

## Materials and methods

The present study has two parts: (I) development and application of a toothbrushing systematics index based on theoretical data and (II) the validation of this index with clinical data from participants before and after being instructed in toothbrushing systematics. The clinical data were obtained from the re-analysis of toothbrushing videos from an earlier study [11], using INTERACT^®^ (Professional Software for Observational Research, Mangold International GmbH, Arnstorf, Germany). The video analysis has been described elsewhere in detail [8]. Both the theoretical data and the clinical analysis resulted in timed-event sequential data [13] which are required to obtain detailed information on the whole brushing process.

### Description of the (clinical) video material and of the video analysis

The video material was obtained from re-analysing videos from a previous randomized controlled clinical trial on the adoption of a toothbrushing technique and systematics [11]. The study was performed according to the guidelines of Good Clinical Practice and the Declaration of Helsinki, and it was approved by the local Independent Ethics Committee of the Justus-Liebig-University Giessen (05/05). The committee also approved the use of these data for the present study (amendment to 05/05). The study details are described elsewhere [11].

In brief, healthy students with no orthodontic appliances and no removable prosthesis, not related to dentistry or medicine, were included into the study (mean age 26.6 years; range 19–42 years). All participants gave written informed consent. The intervention consisted of two verbal instructions of the toothbrushing systematics according to Rateitschak (Fig 1 [3]) either through a leaflet or with a practical demonstration using a model, and home training periods (two weeks each) after the first (T1) and second interventions (T2). All participants were filmed while being aware of this procedure (for discussion of the method see [5,14]) three times (at baseline (BL), T1 and T2). The participants in the instruction group received the first and second instruction after filming at BL and at T1, respectively. In the control group, the participants received an instruction after filming at T2 and were filmed a fourth time after this instruction without any training period (T3).

Two investigators (KW, TW) coded the videos using the software INTERACT^®^. A coding system for representing the continuous timed-events was [15] developed. Videos were coded exhaustively, so every second of the observation session was coded for continuous timed-event behaviour sampling [15,16]. Video analysis parameters, such as sextant and surface or frequency of the changes between areas, were based on the studies from Macgregor and Rugg-Gunn [4,5] and a previously performed video observation study [8]. For analysis, the dentition was divided into areas consisting of the oral, occlusal and vestibular surfaces of each sextant (i.e., the lateral and anterior regions of the upper and lower jaw, resulting in 16 areas, plus the 2 areas of the incisal edges). The parameters of interest were effective brushing time (the time the toothbrush acted on teeth excluding unrelated events, such as spitting out) and the sextant and tooth surface where the toothbrush acted. From these parameters, the number of changes between areas, the number of areas reached and the brushing time for each area was calculated.

Both investigators were carefully trained in all procedures and calibrated. For the calibration 15% of the videos were randomly selected and were independently analysed by both investigators to check for the *inter*-rater agreement. The *intra*-rater agreement was checked through the analysis of 10 randomly selected videos. These videos were analysed twice, once directly after the training period and a second time when two-thirds of the videos had already been analysed. The *inter*- and *intra*-rater agreement calculations were made using INTERACT, according to a previous study: duration events were registered if they overlapped by at least 85% and if the start was within a tolerance of 24 frames per second (= 0.96 seconds). The overlapping selection was deactivated for codes with a duration, and a start tolerance of 2 seconds was set [8]. The *inter*-rater agreement for sextants/surfaces was ĸ = 0.89/0.77. The *intra*-rater agreement for the sextants/surfaces was ĸ = 0.89/0.80 directly after the calibration procedure and ĸ = 0.88/0.77 after analysing 2/3 of the videos.

### Theoretical concept and algorithms of the TSI

#### Theoretical concept

Toothbrushing is considered to be systematic when three conditions are met: (a) completeness (all areas of the dentition are reached) (b) isochronicity (the brushing time is equally distributed between all areas of the dentition), and (c) consistency (avoiding frequent alternations between areas); further brushing time was considered.

#### Behavioural measures

The index is designed for timed-event sequential data. Based on this design two behavioural measures were developed to quantify the degree of systematic brushing.

*Consistency (C)*: The first measure, called C, considers the frequency of changes with the toothbrush between the areas in the mouth in relation to the total brushing duration. As the quotient decreases with increasing systematics, it is subtracted from 1. To consider the completeness of the reached areas, the term is multiplied by the quotient of the reached areas and reachable areas.

$$C=\left(1-\frac{b}{x}\right)\cdot \frac{i}{n}$$

*b =* number of changes between the areas, *x* = total brushing duration in seconds, *i* = reached areas, *n* = total number of reachable areas in the mouth.

Hypothetically, the number of changes can be higher than the duration measured in seconds; in this case, the C-value becomes negative. To avoid negative index-values, the C-value is set to zero in these cases.

*Isochronicity (I)*: The second measure, called I, considers the distribution of the brushing duration by the mean absolute deviation of the relative brushing duration in each reachable area in the mouth; it also indirectly considers the completeness, meaning that the duration in an area is zero, if it is not reached, which leads to a distinct increase of the mean deviation:

$$I=1-\frac{\frac{1}{n}\sum_{i=1}^{n}\left|\frac{d_{i}}{x}-\frac{1}{n}\right|}{2\frac{n-1}{n^{2}}}=1-\frac{n}{2(n-1)}\sum_{i=1}^{n}\left|\frac{d_{i}}{x}-\frac{1}{n}\right|$$

*n* = total number of reachable areas in the mouth, *d*_*i*_ = brushing duration within an area in seconds, *x* = total brushing duration in seconds.

The numerator ($\frac{1}{n}\sum_{i=1}^{n}\left|\frac{d_{i}}{x}-\frac{1}{n}\right|$) is the mean absolute deviation of relative brushing duration in each area, and the denominator is the most possible unequal distribution of brushing duration ($2\frac{n-1}{n^{2}}$). The more evenly and completely the areas are reached, the higher the I-value will be because the term behind the 1 will tend to be zero.

Both measures resulted in an index value between 0 and 1. Values tending towards 0 describe poor toothbrushing systematics, whereas values tending towards 1 describe fine to excellent brushing systematics. The two index values were summed up to a total index value, called the TSI (***T***oothbrushing ***S***ystematics ***I***ndex), which describes the degree of toothbrushing systematics. A TSI-value tending towards 2 describes perfect toothbrushing systematics, whereas a TSI-value tending towards 0 describes non-systematic brushing.

### Development and validation of the index

#### Development with theoretical data

For the first step of the index development, 1280 brushing sequences were computationally generated, based on clinical observation data from earlier studies about toothbrushing behaviour [4,8,11,17] and on the mentioned prerequisites.

Variations of the combination of the following parameters were generated: (A) completeness of brushing (the number of reached areas range from 1 (of 16) = only one area is reached, to 16 (of 16) = complete brushing; an area should reflect one area in the mouth if the dental arches are divided into sextants and surfaces, and if the incisal edges are not included); (B) isochronal brushing (the equal distribution of brushing duration between all areas of the dentition) (C) the number of changes between the areas (from 15 = from a given starting area, each further area is approached only once, to 79 = each area (n = 16) is approached 5 times); and (D) the brushing duration (from 30 s to 480 s).

For parameters A, C and D, the range was defined based on findings from the mentioned studies. Within the defined range, adequate intervals were been chosen. For parameter B, one isochronal and one non-isochronal master sequence were built. For isochronal brushing the total time was simply divided into equal parts depending on how many areas are reached in the defined simulated case. The distribution of the non-isochronal durations between the areas were based on clinically observed distributions in the previously mentioned studies. The total brushing duration was divided into sections with disproportionally high and disproportionally low brushing durations per area (for details, see S1 Table).

**Fig 2:**
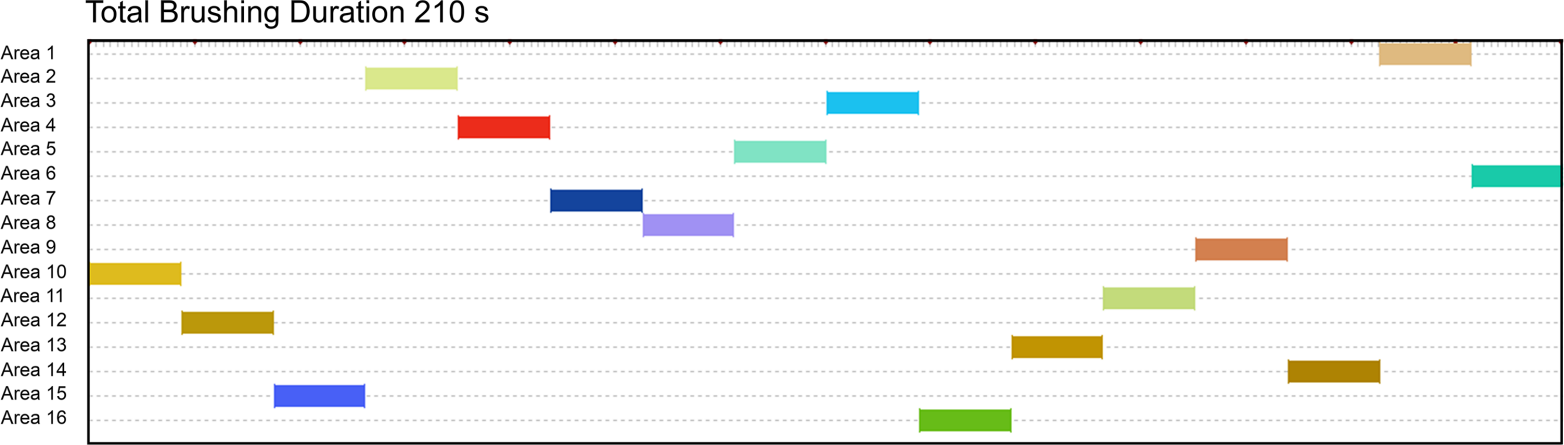
Theoretical data set representing complete, isochronal and consistent brushing. It shows 15 alternations between the areas, with a total brushing duration of 210 seconds; all reachable areas were brushed with an equal brushing duration. Therefore, both I and C values reached their maximum (I = 1 and C = 0.93). The sum is a TSI-value of 1.93, indicating highly systematic brushing. Note that the order in which the areas are reached has no impact. The graph was made with the analysing software INTERACT® (Professional Software for Observational Research, Mangold International GmbH, Arnstorf, Germany).

One example for a theoretical toothbrushing process is shown as a timeline chart in Fig 2. The index values were calculated according to abovementioned measures for each combination of the various parameters.

#### Validation of the toothbrushing systematic index (TSI) with clinical data

The TSI was validated using data from the re-analysed video material from the clinical study described above [11]. This validation was performed in two consecutive steps. In step one, the TSI was calculated only for those videos showing participants who completely (COMP) adopted the Rateitschak systematics which was defined as the gold standard. According to the analysis of the previous study [11], complete adoption was classified when the pre-defined course of brushing was fully completed. These videos were compared to their respective baseline (pre-instruction) videos. This step was performed to determine the maximal achievable values of both C and I when the subjects brushed in a way that was considered the gold standard.

In the second validation step, the TSI was calculated for all participants at T1, T2 and T3 (instruction of the control group), independent of the full adoption of the Rateitschak systematics. This step was performed to estimate how the index mirrors post-instruction changes of brushing behaviour under clinical study conditions (CLIN).

For both steps, COMP and CLIN, the index values were calculated for two conditions (‘all’ and ‘v/o’). In condition ‘all’, all surfaces were analysed; in condition ‘v/o’ only the vestibular and oral surfaces were analysed.

### Statistics

Statistical analysis for the clinical data only was performed with SPSS 24 for Windows (IBM, Armonk, NY, USA). Data from each group were checked for normal distribution (Kolmogorov-Smirnov test). *Intra*-group comparisons between two time points were calculated with paired-samples t-tests (for step CLIN of the validation, a Bonferroni-adjustment was necessary; after the adjustments, the level of significance was: p ≤ 0.008 for the index values and p ≤ 0.002 for the duration values). *Inter*-group comparisons within one time point were calculated with a One-Way ANOVA. All TSI and duration values are presented as the mean ± standard deviation. Unless otherwise noted, the level of significance was set at p ≤ 0.05.

## Results

### TSI theoretical data

#### Isochronal data

**Fig 3:**
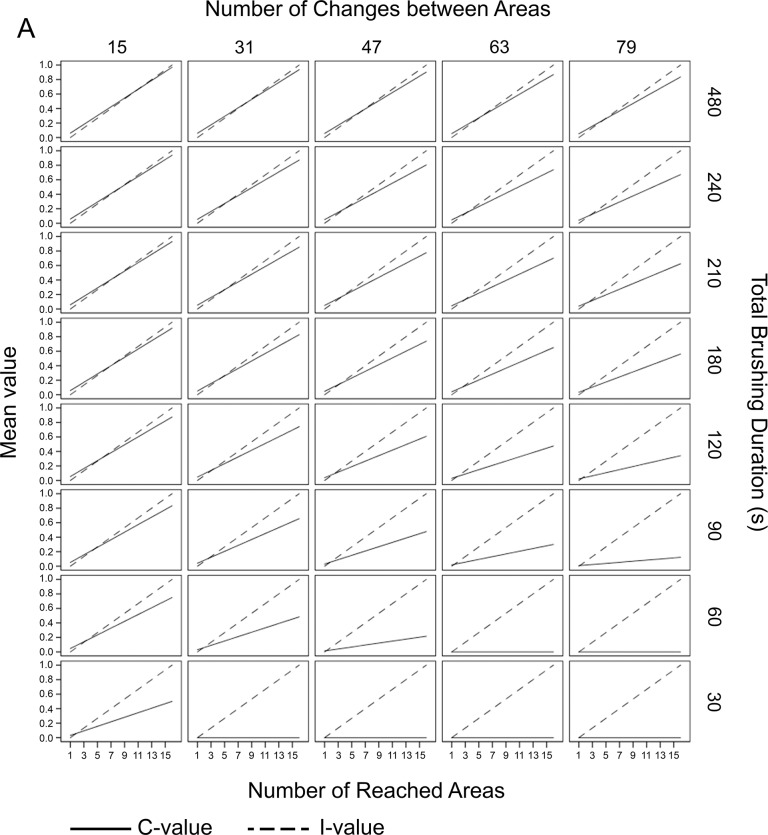
Matrix of isochronal theoretical data. The matrix shows the results of the C- and the I-values of the toothbrushing condition for which all areas were reached for the same duration (isochronal brushing). Negative I-values were replaced by zero. If an absolute isochronal brushing is generated, all I-values are on the diagonal on the graph. The C-value can reach a minimum of 0.0 and a maximum of 0.97, and the I-value can show values between 0.0 and 1.0. The graph clearly shows that even if, in theory, the I-value is high (isochronicity), the C-value can be worse if the changes between the areas are high and the absolute brushing duration is low.

Under the condition that all areas were reached for the same duration, the TSI-values ranged between 0.00 (least systematic) and 1.97 (perfectly systematic). The mean TSI-value was 0.81 ± 0.51. The C-values ranged between 0.00 and 0.97 (mean: 0.31 ± 0.26) and between 0.00 and 1.00 (mean: 0.50 ± 0.31) for I. A matrix showing all calculated C- and I-values for all conditions is provided in Fig 3.

#### Non-isochronal data

**Fig 4:**
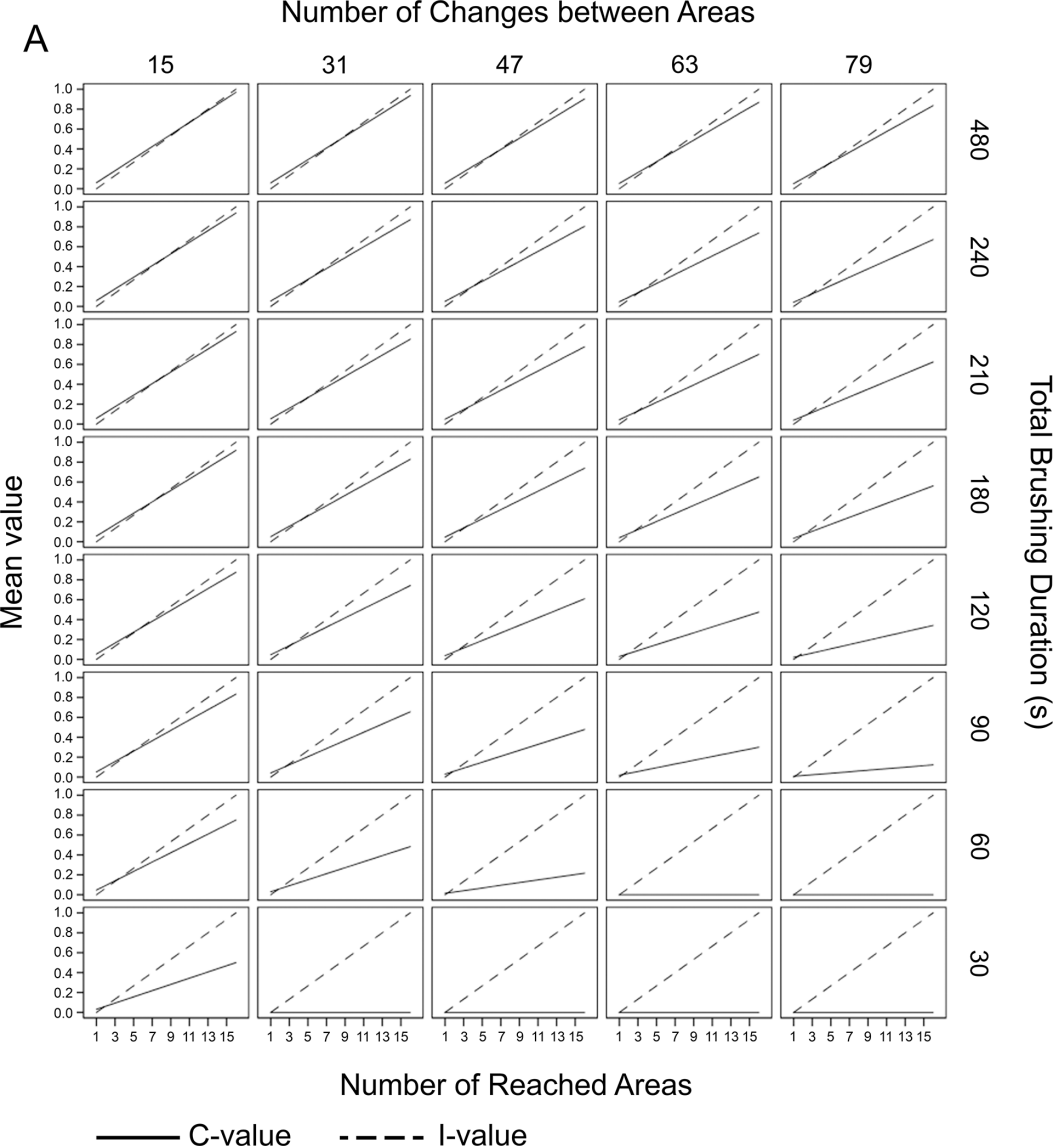
Matrix of non-isochronal theoretical data. Same matrix as shown in Fig 3 except the brushing was non-isochronal; the areas were reached for different durations. Negative I-values were replaced by zero. There was no change in C-value; however, the maximum I-value decreased to 0.8, thus reflecting the necessity of isochronicity for a very high I-value.

Under the condition that in which the areas are reached with different durations, the TSI-values ranged between 0.00 and 1.77. The mean TSI-value was 0.78 ± 0.47. For the C-value no difference to isochronal data was found, the values ranged between 0.00 and 0.97 (mean: 0.31 ± 0.26). The I-value decreased and ranged between 0.00 and 0.80 (mean: 0.46 ± 0.26). A matrix showing all calculated C- and I-values for all conditions is provided in Fig 4.

### Validation of TSI using clinical data

**Fig 5:**
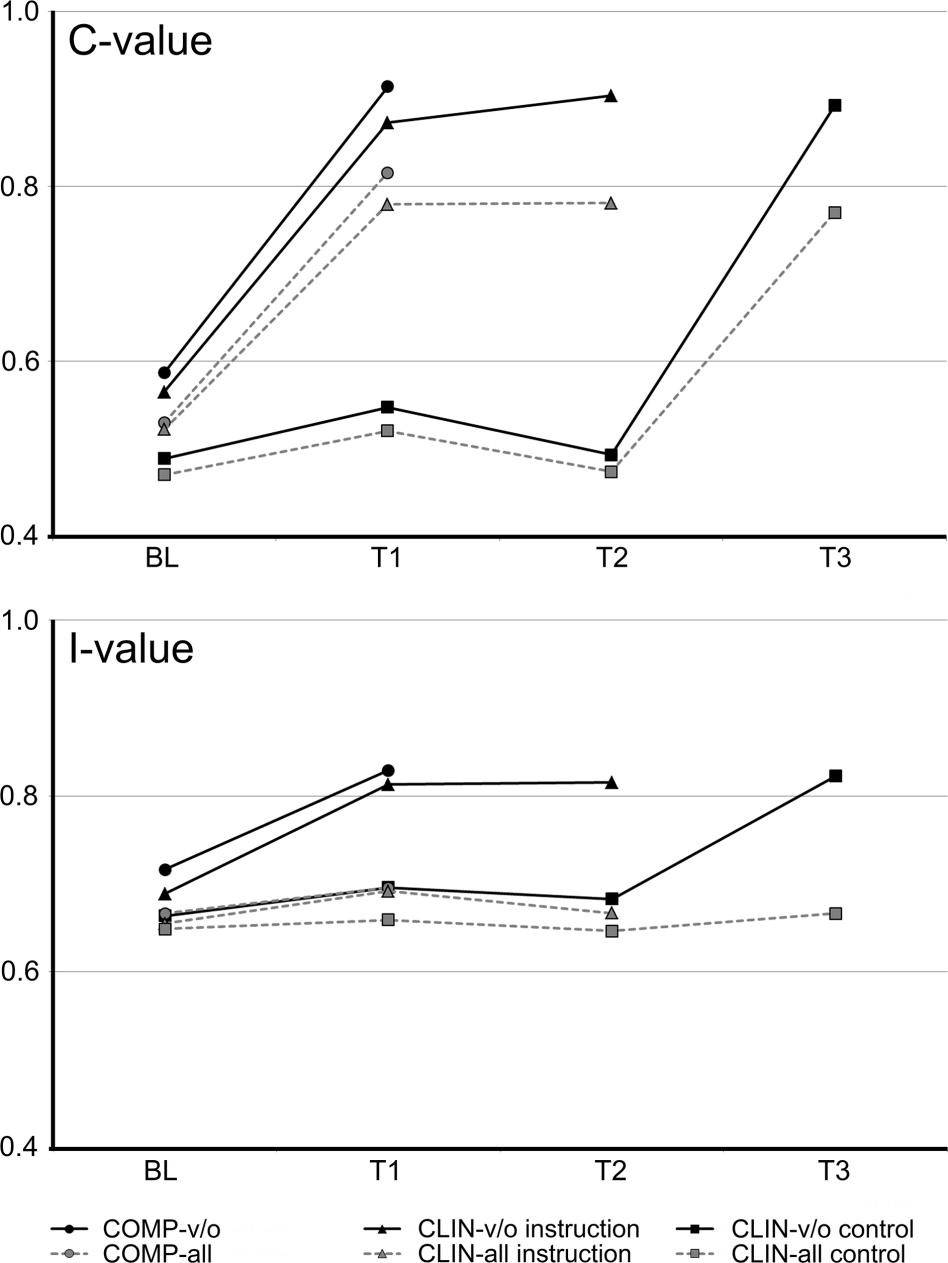
Clinical validation of TSI. Results of the C-value and I-value calculation of the clinical validation of the TSI. Dashed lines show the results if all areas are considered and solid lines show the results of the analysis of vestibular and oral areas. The graphs clearly show that the index has a higher discrimination power if only the vestibular and oral surfaces are analysed, and that the I-value shows no change due to instruction if all areas are analysed. The instruction in the last appointment of the control group led to the same increase in index values as the instructions in the other group. Except for the I-values, which were calculated to analyse ‘all’ surfaces, the values of the COMP analysis showed a tendency towards higher index values compared with the CLIN analysis. Note that the x-axis does not intersect the y-axis at the zero position. For clarity, no standard deviations are given; the mean values and standard deviations are given either in the text or in Table 1.

Data for the C- and I-values of CLIN under both conditions ‘all’ and ‘v/o’ are given in Table 1 and of COMP and CLIN in Fig 5. The results for the total brushing duration and the brushing duration on oral, vestibular and occlusal surfaces at different time points are given in Table 2.

**Table 1:** Clinical validation data.

	Baseline (BL)			After first			After second			Control group		
				instruction (T1)			instruction (T2)			after instruction (T3)		
**CLIN-all**	C	I	TSI	C	I	TSI	C	I	TSI	C	I	TSI
Control	^a^0.47^A^	^a^0.65^A^	^a^1.12^A^	^a^0.52^A^	^a^0.66^A^	^a^1.18^A^	^a^0.47^A^	^a^0.65^A^	^a^1.12^A^	^b^0.77	^a^0.67	^b^1.44
	(0.14)	(0.10)	(0.21)	(0.11)	(0.11)	(0.20)	(0.17)	(0.10)	(0.24)	(0.10)	(0.04)	(0.13)
instruction	^a^0.52^A^	^a^0.66^A^	^a^1.18^A^	^b^0.78^B^	^a^0.69^A^	^b^1.47^B^	^b^0.78^B^	^a^0.67^A^	^b^1.45^B^			
	(0.12)	(0.09)	(0.18)	(0.08)	(0.06)	(0.12)	(0.11)	(0.08)	(0.17)			
**CLIN-v/o**	C	I	TSI	C	I	TSI	C	I	TSI	C	I	TSI
Control	^a^0.49^A^	^a^0.66^A^	^a^1.15^A^	^a^0.55^A^	^a^0.70^A^	^a^1.24^A^	^a^0.49^A^	^a^0.68^A^	^a^1.18^A^	^b^0.89	^b^0.82	^b^1.72
	(0.18)	(0.13)	(0.29)	(0.17)	(0.15)	(0.28)	(0.22)	(0.15)	(0.34)	(0.10)	(0.05)	(0.14)
instruction	^a^0.56^A^	^a^0.69^A^	^a^1.25^A^	^b^0.87^B^	^b^0.81^B^	^b^1.69^B^	^b^0.90^B^	^b^0.82^B^	^b^1.72^B^			
	(0.16)	(0.14)	(0.27)	(0.08)	(0.07)	(0.14)	(0.08)	(0.07)	(0.13)			

Display of the means (SD) of the C-, I- and TSI-values of the validation step CLIN under the condition ‘all’ (including all areas) and the condition ‘v/o’ (analysing only the vestibular and oral surfaces). Note that under the condition CLIN-all, the I-value did not change after the instruction but did so under the CLIN-v/o condition. Lowercase letters indicate significant differences between time points within one group and parameter, and uppercase letters indicate significant differences between the instruction and control group at the same time point within one parameter.

**Table 2:** Brushing duration.

	Baseline (BL)				after first instruction (T1)				after second instruction (T2)			
	Total	vest.	occl.	oral	Total	vest.	occl.	oral	Total	vest.	occl.	oral
*CLIN-Control*	^a^126.6^A^	^a^53.8^A^	^a^44.5^A^	^a^28.2^A^	^a^146.4^A^	^a^64.0^A^	^a^44.1^A^	^a^38.3^A^	^a^125.0^A^	^a^55.3^A^	^a^36.4^A^	^a^33.3^A^
	(58.6)	(31.6)	(26.1)	(22.8)	(60.9)	(29.9)	(29.1)	(25.7)	(53.2)	(24.1)	(22.9)	(28.0)
*CLIN-Instruction*	^a^142.5^A^	^a^71.4^A^	^a^37.0^A^	^a^34.0^A^	^b^236.1^B^	^b^101.9^B^	^a,b^25.1^B^	^b^109.1^B^	^c^294.8^B^	^c^128.5^B^	^b^21.6^B^	^c^144.7^B^
	(53.8)	(32.3)	(23.9)	(25.8)	(103.8)	(47.3)	(19.0)	(58.9)	(127.5)	(61.9)	(16.8)	(71.2)
*CLIN—Control* after Instr. (T3)					^b^272.3	^b^116.3	^b^19.7	^b^136.4				
					(130.4)	(62.2)	(12.1)	(72.1)				
*COMP*	^a^146.7	^a^72.0	^a^34.0	^a^40.6	^b^250.6	^b^107.3	^b^22.6	^b^120.7				
	(48.4)	(30.9)	(23.1)	(25.5)	(109.7)	(51.5)	(11.3)	(61.7)				

The brushing duration is shown in seconds (mean (SD)) for the various time points in the control and instruction groups under clinical conditions (CLIN) and under the condition that the Rateitschak systematic was fully adopted (COMP). Statistical significance for the comparison between the control and instruction groups within one time point is marked by different uppercase letters; for the comparison between time points within one group, it is marked by different lowercase letters. Note that “CLIN-Control after instruction” was also compared to all other time points of “CLIN-Control”. The comparison between the instruction group after the first instruction and the control group after the instruction revealed no significant difference.

#### COMP (participants who completely adopted the systematics according to Rateitschak–analysis of clinical observational data)

A total of 26 participants presented with complete adoption of the systematics after the first instruction in the instruction group (T1) and after instruction in the control group (T3).

*COMP-all*: The TSI at baseline was 1.20 ± 0.18 (C-value 0.53 ± 0.12, I-value 0.67 ± 0.10). After the complete adoption of the toothbrushing sequence, the TSI increased significantly (1.51 ± 0.09; p≤0.001) as did the C-value (0.82 ± 0.06; p≤0.001); the I-value, however, remained constant (0.70 ± 0.05; n.s.).

*COMP-v/o*: The TSI at baseline was 1.30 ± 0.26 (C-value 0.59 ± 0.15, I-value 0.72 ± 0.13). After the complete adoption of the toothbrushing sequence, all values increased significantly (TSI 1.74 ± 0.09, C-value 0.91 ± 0.04, I-value 0.83 ± 0.06; all p≤0.001).

#### CLIN (analysis of clinical observational data)

In total 59 participants were included (control n = 17; instruction n = 42).

*CLIN-all*: At BL, the intervention and control groups showed similar TSI-values (p > 0.05). In the uninstructed control group, the TSI-value remained constant at T1 and T2 (p > 0.05); when this group received the instruction at the end of the study (T3), the TSI-value increased significantly compared to T1 and T2 (p ≤ 0.001). In the intervention group, the TSI increased significantly after the first instruction (BL to T1, p ≤ 0.001) but remained constant after the second instruction (T1 to T2, p > 0.05). Between the intervention and control groups, significant differences were found at T1 and T2 (p ≤ 0.001). No significant differences were found between the control group after the intervention and the intervention group, independent of the time point. The C-value showed the same behaviour as the TSI-values with the same significances between groups and time points. However, the I-value remained constant, and no significant differences were found between the groups and at no time point.

*CLIN-v/o*: Both groups showed at BL a similar TSI (p > 0.05). In the control group, the TSI-value remained constant at T1 and T2 (p > 0.05). The intervention in the control group (T3) significantly increased its TSI-value compared to those of T1 and T2 (p ≤ 0.001). In the intervention group, the TSI significantly increased from BL to T1 (p ≤ 0.001) but remained constant from T1 to T2 (p > 0.05). As shown for CLIN-all, significant differences were found at T1 and T2 for the control and intervention groups (p ≤ 0.001). No significant difference was found between the control group after the intervention and the intervention group, independent of the time point. In contrast to the values after the analysis of all areas, both the C- and the I-values reflect the results of the TSI.

## Discussion

Although there are suggestions regarding brushing systematics (e.g., Rateitschak), a validated concept for an analysis of its components has not yet been published. The study thus presents first-time a theoretical framework regarding the plausibility and practical logic.

Three aspects directly related to systematics were addressed: (a) completeness, (b) isochronicity and (c) consistency. Completeness is the most obvious component of systematic brushing because plaque accumulates on tooth surfaces that a toothbrush does not reach. In addition to this obvious factor, isochronicity was chosen as another component. Whether all areas should be brushed with approximately the same time, however, could be a matter of consideration. In the absence of oral hygiene, plaque accumulates unevenly throughout the dentition [18,19], and calculus is most prevalent on the oral surfaces of the lower anterior teeth. Further, bridgework, removable prostheses or mal-positioned teeth constitute individual risk factors for plaque accumulation. These aspects may indicate differentiated cleaning needs in different areas or different individuals. As a general rule, however, and for didactical reasons, recommending isochronal brushing seems to be reasonable. A similar rule holds true for the consistency of brushing. In theory, all regions of the dentition can be sufficiently cleaned as soon as all teeth are reached and brushed for a sufficiently long period, even if a subject frequently moves between areas. It would, however, surely increase an individual’s self-control to concentrate on a region until it is sufficiently cleaned before proceeding to another.

What has not been included is the order of a brushing sequence. Thus far, there is no evidence or plausible argument that one specific order within a brushing sequence is superior to another. In fact, it has been shown that there was no difference in the reduction of plaque scores after a highly standardised systematic brushing when subjects started at the lingual compared to starting at the buccal aspect [20].

The theoretical data considered variations of these single aspects in a clinically meaningful range, and the matrices depicted in Figs 3 and 4 clearly show that the chosen algorithms produce index values that reflect the components of brushing systematics in a reasonable manner.

The C-value is sensitive to the reached areas, which means that even a long brushing duration with few changes between areas cannot result in high index values when not all areas are reached. It is further modified by brushing duration as the value decreases with short brushing time such as 90 s and below, even when all areas are reached and few changes occur. The number of changes impacts the index value when the brushing duration is shorter. Non-isochronal brushing behaviour, however, has no impact on the C-value; the graphs in both matrices are identical.

In contrast, this aspect modulates the I-value. In the theoretical isochronal data, the I-value is affected only by the number of areas reached. It is therefore synergistic with the C-value boosting the impact of not reaching all areas in the combined index value, which is meaningful in the clinical context. Further, it modifies the relatively distinct impact of brushing time in the C-value in a potentially meaningful manner. In the non-isochronal data, however, the maximum index value cannot be reached as long as isochronicity is not fulfilled. From a clinical viewpoint, isochronicity is relevant but should not be handled in too strict manner which is reflected in the moderate modulation effect of the I-value. As both values reflect equally important aspects, the single values have been weighted equally.

Theoretical data can demonstrate the principal behaviour of the components of the index but are schematic and can mimic the true behaviour of subjects only in a limited manner. Therefore, the validation process used clinical data.

The TSI was used with these data under two conditions: including or excluding the occlusal surfaces (Table 1). Oral hygiene instructions mostly include brushing the occlusal surfaces, but from a clinical viewpoint it might be questioned whether it is a meaningful recommendation for the full occluding dentition to brush these areas extensively. On occlusal planes, plaque is hardly found even in subjects with poor hygiene, which is also reflected in the fact that established plaque indices do not include these areas. Therefore, it should be contemplated whether the occlusal surfaces should be brushed for a shorter time than the other surfaces. The Rateitschak systematics considers this prospect in that it emphasises the oral and vestibular areas, whereas the occlusal sites are placed at the end of the brushing course. Thus, isochronicity in particular would potentially constitute a serious flaw within the concept of the TSI when occlusal surfaces are included, which became obvious when the abovementioned two conditions of analysis were compared; the respective aspects are discussed below.

Clearly, the new index should produce high index values in subjects who have fully adopted the Rateitschak systematics (COMP) and should reflect their much less systematic brushing behaviour at baseline, with distinctly lower index values. For the full analysis that included all surfaces, however, this result was only partially achieved. As expected, the baseline values were much lower than the post instruction values; however the latter did not approximate the maximum, primarily due to the I-value, which did not increase compared to baseline. When the occlusal surfaces were excluded, however, the C-value, and particularly the I-value, increased considerably, ending up at values of 0.91±0.04 and 0.83±0.06, respectively, and for a TSI value of 1.74.±0.09. This difference between the full and partial analyses is clearly explained by the fact that the subjects were not instructed to brush the occlusal surfaces for as long a duration as they brushed the other areas, resulting in much shorter occlusal brushing times compared to oral and vestibular surfaces. The reason for the reduced discriminative power of the I-value (Fig 5) under the condition ‘all’ is, therefore, primarily mathematically located; it is plausible that an intended unequal brushing decreases the I-value. However, the findings also demonstrate the importance of the I-value in general, which was less distinct in the theoretical data. Even if the occlusal surfaces were excluded, a significant higher TSI value in those participants who complete adopted the systematic (COMP) compared to the remaining participants was found (data not shown); however, the maximum values were still not reached. Instead, the mean TSI of 1.74±0.09 corresponds well to the maximum of the TSI of 1.77 in the theoretical non-isochronal data. Indeed, though having the Rateitschak systematics fully adopted, the subjects brushed longer on oral surfaces than on vestibular surfaces (Table 2), thus confirming the non-isochronal brushing indicated by the I-value, because the participants were not instructed to brush all areas with an equal duration, but were only instructed to reach all areas. Full adoption of the systematic was scored regardless of the brushing time per area.

The new index should also be able to demonstrate effects beyond the optimal adoption of a brushing systematics (second validation step). When all results after the instruction were evaluated, this expectation was clearly demonstrated. Similar to the effects seen in the group that fully adopted the Rateitschak systematics, the discrimination power seems to be better when the occlusal surfaces were excluded (Fig 5). The values clearly and significantly increased after intervention but remained constant in the control group at any time, showing an instructional related improvement in brushing systematics but also a constant brushing behaviour of the participants and a robustness of the index under non-instruction conditions. The results of the analyses clearly show that the first instruction was quite effective. The second instruction (remotivation), however, rather consolidates the effect of the first one but does not bring a significant additional effect.

The intention of this index is to use it in observational studies considering the individual brushing behaviour and with oral hygiene instruction strategies. It could be used to analyse how effective and, in particular, how sustainable intervention strategies are. An index needs to be valid and usable in multiple settings. Thus, the suggested TSI can be easily adapted to different requirements. It can be calculated from all kinds of observational studies and methods of analyses, as long as quantitative data on the total brushing duration, the brushing duration per area, the movement frequency between areas and the number of reached areas are provided. Further, it can be used with different definitions of areas (e.g. quadrants instead of sextants) or modified for partially dentate subjects by adapting the number of reachable areas in the algorithms. Though not directly related to systematics, a sufficient brushing duration can also be considered a basic requirement for plaque control. When brushing with a manual toothbrush is performed by a professional person ensuring an effective brushing technique, the percentage reduction in the plaque scores (Silness & Loe plaque index) was 54, 67 and 74% after 1, 2 and 3 minutes, respectively, showing that duration is a significant factor for efficacy [21]. Therefore, if desired, the absolute brushing duration could be added as a third value (for example as a ratio between performed brushing duration and recommended brushing duration).

In conclusion, the suggested new TS-index can quantify changes in toothbrushing systematics in a valid manner. It consists of two index values that describe different parameters of systematic brushing behaviour in detail and are combined to an overall TSI score. The TSI is independent from predefined orders of brushing sequences and is therefore able to cover a variety of clinically meaningful variations of systematic brushing. Further observational studies need to both determine whether the C- and I-values are related to specific aspects of behaviour change and investigate their relative roles in the combined TS-index. Finally, studies relating the TSI to the plaque control efficacy scores are clearly necessary.

## Supporting information

S1 Table Excel sheet of theoretical data for index development. (XLSX)
